# Epidemiological and molecular analysis of the Chikungunya virus outbreak in the state of Rondônia

**DOI:** 10.1007/s15010-026-02773-3

**Published:** 2026-04-24

**Authors:** Karolaine Santos Teixeira, Tárcio Peixoto Roca, Ana Maisa Passos-Silva, Edilene Pereira Pimentel, Jackson Alves da Silva Queiroz, Lourdes Maria Pinheiro Borzacov, Jansen Fernandes Medeiros, Juan Miguel Villalobos Salcedo, Deusilene Vieira

**Affiliations:** 1 Laboratório de Virologia Molecular, Fundação Oswaldo Cruz Rondônia - FIOCRUZ/RO, Rua da beira, 7176, Bairro Lagoa, Porto Velho, Rondônia 76812-245 Brazil; 2 Programa de Pós-Graduação Em Biologia Experimental, Universidade Federal de Rondônia e Fundação Oswaldo Cruz Rondônia – UNIR/FIOCRUZ/RO, Porto Velho, 76801-974 Brazil; 3 Centro de Pesquisa em Medicina Tropical – CEPEM, Porto Velho, 76812-329 Brazil; 4 Universidade Federal de Rondônia - UNIR, Porto Velho, 76801-974 Brazil; 5 Laboratório de Entomologia, Fundação Oswaldo Cruz Rondônia - FIOCRUZ/RO, Porto Velho, RO Brazil

**Keywords:** Arboviruses, Chikungunya virus, Epidemiological surveillance, Amazon

## Abstract

**Purpose:**

The Chikungunya virus (CHIKV) is an arbovirus transmitted by hematophagous arthropods, widely distributed in tropical and subtropical regionsAQ1. In Brazil, its introduction in 2014 marked the beginning of a significant expansion, becoming a major public health problem.

**Methods:**

This work consists of a cross-sectional study conducted from October 2024 to July 2025, in which 830 samples from patients with acute febrile illness and negative diagnosis for malaria were evaluatedAQ2. The samples were tested for dengue, Zika, and chikungunya and subjected to duplex RT-qPCR to detect Mayaro and Oropouche.

**Results:**

Of the samples analyzed, 5,18% (43/830) tested positive for some arbovirus investigated in this cohort, of which 53,49% (23/43) were detectable for CHIKV. The phylogenetic analysis showed that all samples belonged to the East/Central/South African (ECSA) genotype, demonstrating its circulation in the region.

**Conclusions:**

The findings reinforce the need for continuous molecular surveillance to monitor the circulation of medically important arboviruses in the Amazon region.

## Introduction

Arboviruses are viruses transmitted by hematophagous arthropod vectors that are widespread in tropical and subtropical regions, with generally seasonal circulation [1, 2]. Among the most medically relevant arboviruses, three main genera stand out: *Orthobunyavirus*, which includes Oropouche virus (OROV) [3]; *Orthoflavivirus*, represented by Dengue (DENV), Zika (ZIKV), and Yellow Fever (YF) viruses [4–6]; and *Alphavirus, to which* Mayaro (MAYV) and Chikungunya (CHIKV) [7–9] viruses belong.Chikungunya fever is an acute febrile disease caused by the Chikungunya virus (CHIKV), belonging to the *Togaviridae* family [10, 11].

Since its identification in 1952, CHIKV has demonstrated broad geographic dissemination, causing outbreaks and epidemics across continents such as Asia, Africa, North America, and South America [12]. In Brazil, the virus was first detected in 2014 in the Northeast region, marking the beginning of its indigenous circulation in the country [13–15]. Since then, there has been a progressive increase in the incidence of the disease, especially in the northeastern states, where CHIKV remains in endemic circulation. This situation has expanded to other regions, including the Western Amazon, where the state of Rondônia has reported outbreaks and confirmed cases of local transmission, highlighting the active circulation of the virus and the need for continued surveillance [19].

In addition to its growing impact on public health, Chikungunya fever poses considerable diagnostic challenges due to its clinical similarities with other arboviruses, such as dengue, Zika, Mayaro, and Oropouche. The clinical picture is characterized by high fever, rash, myalgia, and intense polyarthralgia, and can progress to a chronic phase marked by persistent joint pain, compromising patients' quality of life and overwhelming health services [17, 18]. Currently, there are no specific antiviral therapies or licensed vaccines to prevent the infection, making vector control the primary mitigation strategy [15]. In this context, it is essential to understand the epidemiological, clinical, and molecular aspects of the Chikungunya virus in the Amazon region, where environmental, climatic, and socioeconomic factors can favor the maintenance and intensification of viral transmission. Therefore, the objective of this study was to analyze the molecular epidemiology of the reemergence of the Chikungunya virus in the state of Rondônia.

## Methods

### Ethical aspects

This research was approved by the Research Ethics Committee of the Tropical Medicine Research Center of Rondônia-CEPEM/RO under number CAAE 74466123.0.0000.0011, and informed consent was obtained from all participants. The research procedures were carried out according to the ethical principles stipulated by the 1975 World Medical Assembly and the Brazilian Ministry of Health and followed the standards established in Resolution Nº. 466, December 12, 2012.

### Study site and biological samples

This study is characterized as a cross-sectional observational study, based on the laboratory surveillance of patients with acute febrile syndrome. Individuals were recruited by convenience between October 2024 and July 2025 in three different health units located in different regions of the municipality of Porto Velho (Rondônia, Brazil) and one unit in the municipality of Alto Alegre dos Parecis (Rondônia, Brazil). The inclusion criteria were individuals with acute febrile symptoms, presenting symptoms for 1 to 7 days, and a microscopy result negative for *Plasmodium sp*.

### Viral RNA extraction

Serum samples collected from participants underwent nucleic acid extraction using the EXTRACTA KIT GOLD – Viral DNA/RNA Kit (MVXA-PV96 B/W Gold) in the EXTRACTA 32 automated DNA and RNA extractor (LOCCUS, São Paulo, Brazil). The procedure was performed according to the manufacturer's instructions, with a final elution volume of 70 µL.

### RT-qPCR for arbovirus detection

The extracted material was subjected to RT-qPCR screening for ZIKV, CHIKV, and DENV serotypes 1, 2, 3, and 4 using the ZC D-Typing Bio-manguinhos molecular kit (Instituto de Tecnologia em Immunobiológicos Bio-manguinhos), according to the manufacturer's instructions. For the detection of MAYV and OROV, the reaction was performed following the protocol described by Naveca et al. 2017. Reactions were performed on the [22] QuantStudio™ 7 Pro Real-Time PCR System (Applied Biosystems).

### Genomic sequencing for Chikungunya

Detectable samples with Ct < 30 for CHIKV were subjected to conventional PCR using the *primers* ALPV_JQ_R ('5 GTNGTRTCRAANCCDATCCARTA 3') and ALPV_JQ_F ('5 AATGACCATGCTAAYGCYAGAG 3') that amplify a 439 base pair (bp) product corresponding to the nsP1 region of the viral genome [23]. The PCR product was sequenced by the automated Sanger method in partnership with Fiocruz Technology Platforms Network (Unit RPT01N) using the SeqStudio equipment. Genetic Analyzer (Applied Biosystems, Massachusetts, USA). The sequencing reaction was performed using the BigDye Terminator v1.1 Cycle kit Sequencing Kit (Applied Biosystems ™, CA, USA) and the reaction product was purified using BigDye XTerminator Kit (Applied Biosystems ™, CA, USA). Electropherograms were analyzed and edited using MEGA v.11.0 software [24].

### Analysis of phylogenetics

Complete CHIKV sequences (n = 71) were retrieved from the GenBank database hosted by the National Center for Biotechnology Information (NCBI) [25] and aligned with the study sequences using MAFFT v7 software [26]. The general time-reversible (GTR) substitution model, which includes gamma-distributed rate variation among sites (G4) and empirical base frequencies (F), was selected based on the best Bayesian Information Criterion (BIC) score as determined by ModelFinder [27]. The phylogenetic tree was reconstructed using the maximum likelihood (ML) method with IQ-TREE v2.2.2.6 [28]. Bootstrap support values were calculated with 1000 replicates.

### Statistical analysis

Prism v8.0 and JASP (version 0.95) software. Categorical variables were expressed as absolute and relative frequencies, while continuous variables were presented as mean and standard deviation or median and interquartile range, according to the data distribution. Normality was assessed using the Shapiro–Wilk test. The association between categorical variables was verified using the chi-squared test or Fisher's exact test, when appropriate. To estimate the strength of the associations, odds ratios were calculated. ratio, OR) with 95% confidence intervals. *P*-values less than 0.05 were considered statistically significant.

## Results

Between October 2024 and July 2025, 830 samples from individuals with acute febrile conditions and a negative diagnosis for malaria were evaluated. The study population consisted of 54% (449/830) males and 46% (381/830) females, with an age range between 18 and 65 years and a mean age of 42 years. The evaluation of symptoms allowed identifying that the most prevalent symptoms were headache in 89.04% (739/830), back pain in 77.35% (642/830), myalgia in 69.04% (573/830), arthritis in 68.67% (570/830), retroorbital pain in 56.27% (467/830), nausea in 51.33% (426/830), intense arthralgia in 29.40% (244/830), vomiting in 26.87% (223/830), rash in 9.88% (82/830), petechiae in 7.71% (64/830), and conjunctivitis in 5.42% (45/830).

**Fig. 1:**
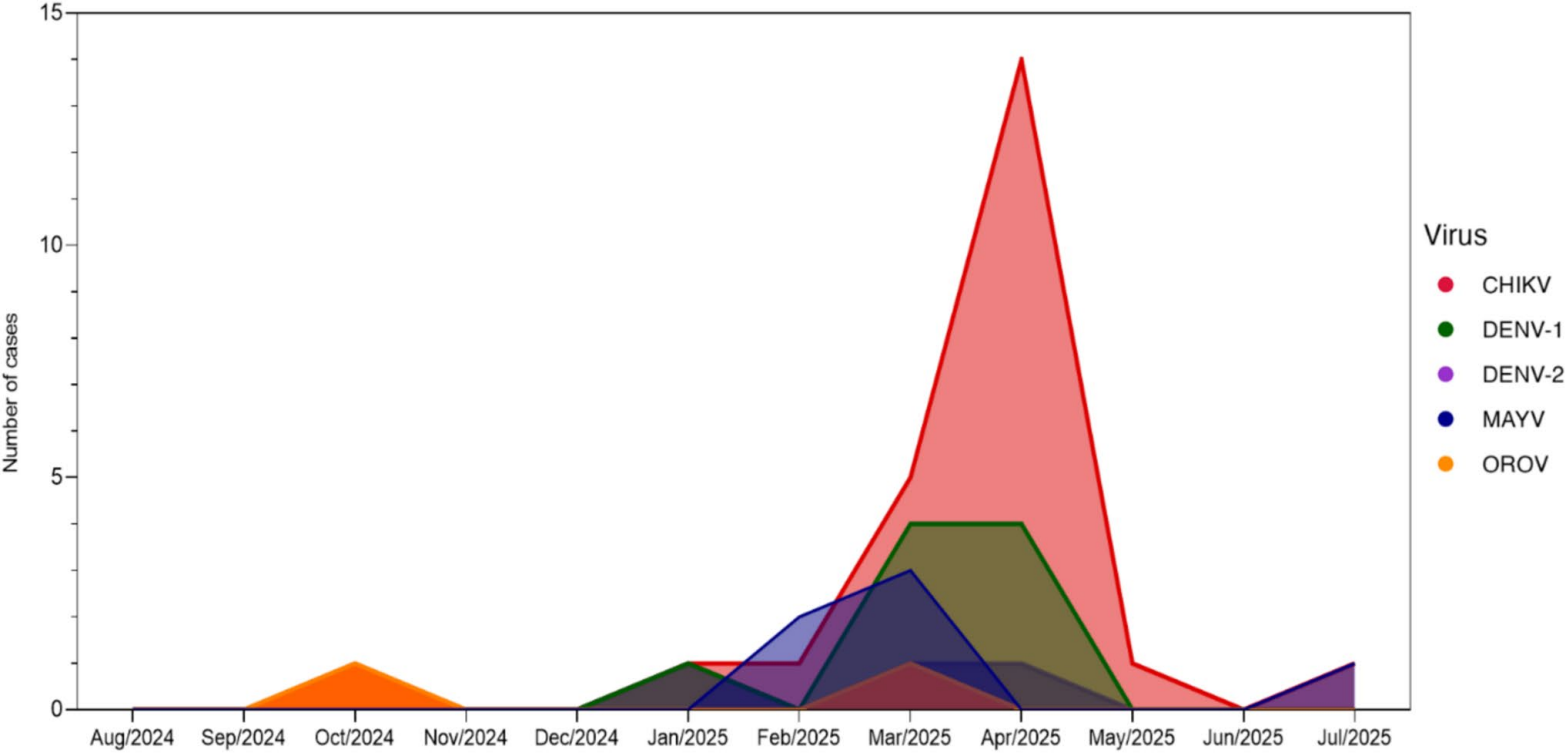
Characterization of detectable cases over the months of evaluation of this study. Legend: Distribution of detectable cases of arboviruses over the months, with each virus represented by a different color for easier visualization

The overall prevalence of detectable arboviruses was 5.18% (95% CI: 3.67–6.69). CHIKV presented the highest prevalence (2.77%; 95% CI: 1.65–3.89), followed by DENV-1 (1.08%; 95% CI: 0.38–1.79), MAYV (0.72%; 95% CI: 0.15–1.30), DENV-2 (0.36%; 95% CI: 0.00–0.77), and OROV (0.24%; 95% CI: 0.00–0.57). Among the detectable cases, 53.49% (23/43) tested positive for CHIKV, 20.93% (9/43) for DENV-1, 13.95% (6/43) for MAYV, 6.98% (3/43) for DENV-2, and 4.65% (2/43) for OROV. No positivity was observed for the other targets evaluated. The temporal distribution of detectable cases over the months is shown in Fig. 1.

**Fig. 2:**
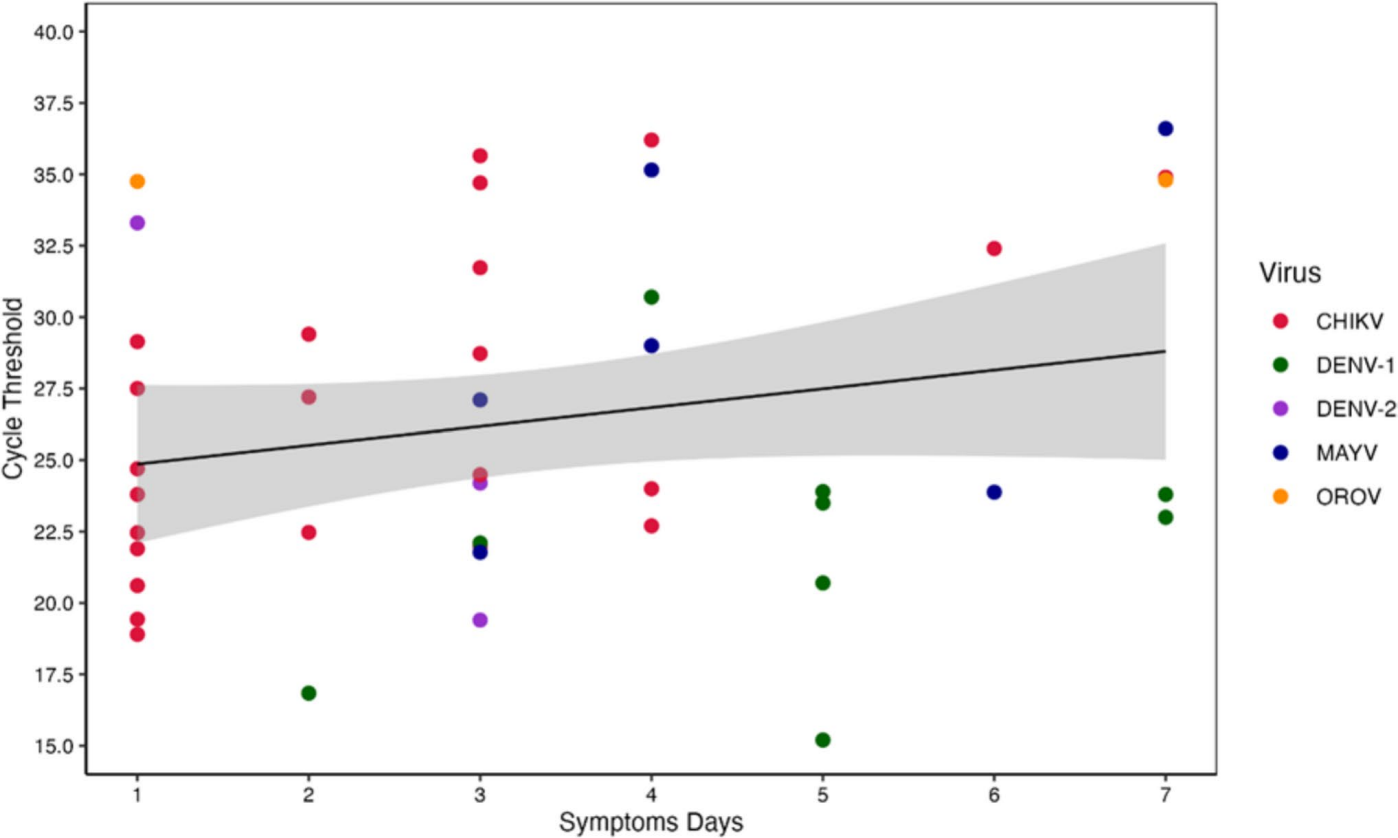
Correlation between the Ct value and the number of days of symptoms of positive individuals. Legend: Scatterplot showing the *Spearman correlation (rho* = 0.225) between days of symptoms and Ct of detectable samples for CHIKV, DENV-1, DENV-2, MAYV, and OROV. The analysis did not show a statistically significant result (p-value = 0.1462)

Positive samples showed a wide dispersion of Ct values, with an interquartile median of 24,3. Symptom days had a mean of 3 days (SD = 2,1) (Fig. 2). Most CHIKV infections occurred between days 1 and 4 of symptoms. These results indicate a high viral load in the early stages, a typical characteristic of CHIKV infections.

**Fig. 3:**
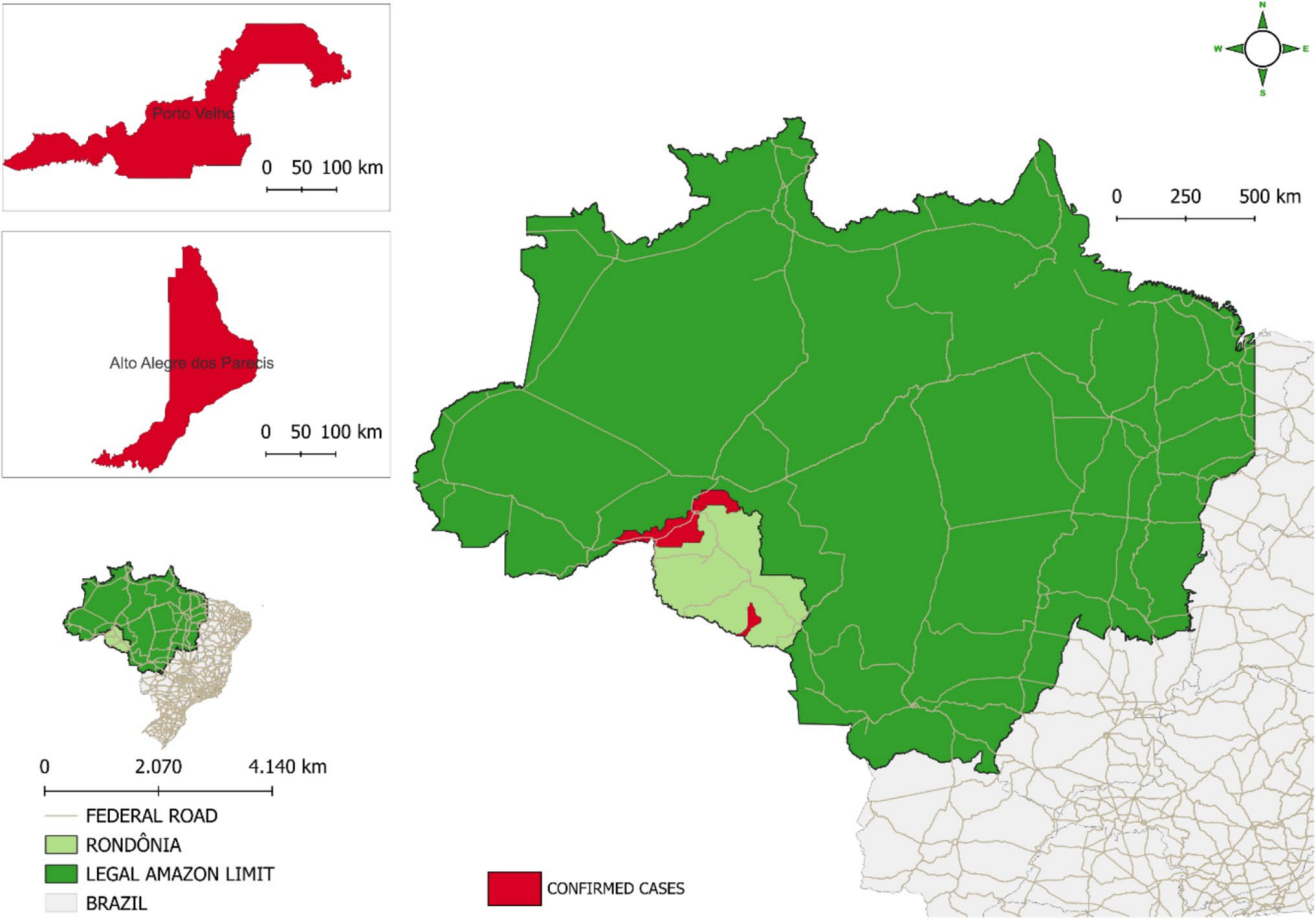
Distribution map of confirmed Chikungunya cases in Rondônia and the federal highway route. Legend: Geographic distribution map showing the locations in the state of Rondônia covered by patients with molecular positivity for Chikungunya virus. Cities with identified cases are highlighted in red; the border of the Legal Amazon (in green) and the federal highway (dark gray line) demonstrate possible routes of entry of the virus into the state

**Fig. 4:**
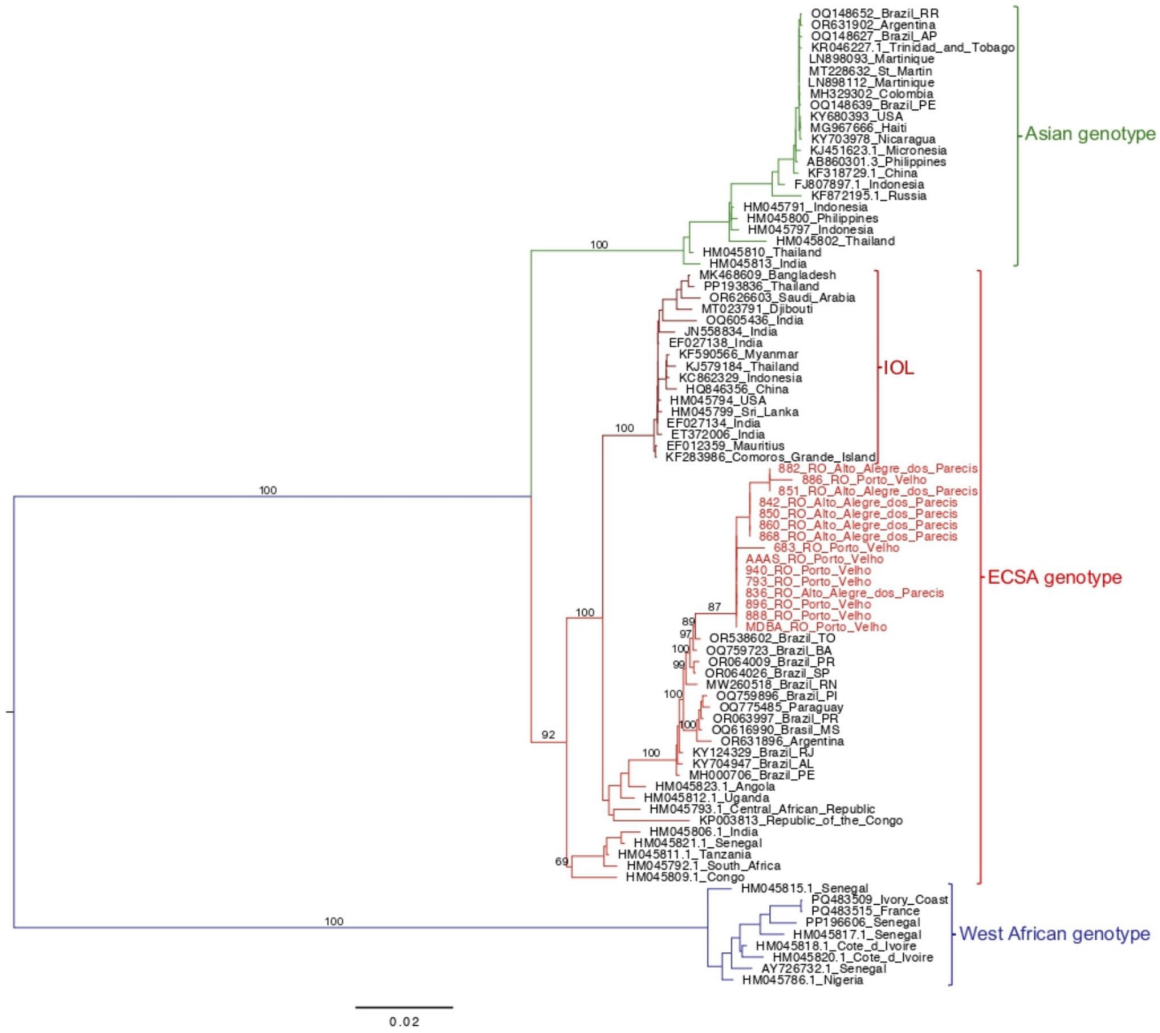
Phylogenetic analysis of samples detectable for CHIKV. Legend: ML tree containing 71 complete CHIKV genomes retrieved from Genbank and 15 sequenced samples from the study. The clades of the Asian, East/Central/South African (ECSA), Indian Ocean Lineage (IOL), and West African genotypes of CHIKV are highlighted in color, and the taxa of the study samples are in red. Bootstrap values are contained in the main branches

Regarding the geographic distribution, it was observed that 57% (13/23) of the patients were from the capital, Porto Velho, while 43% (10/23) resided in Alto Alegre dos Parecis, a municipality located in the southern region of the state of Rondônia (Fig. 4). It is noteworthy that the first two cases identified in Porto Velho refer to patients who reported a history of travel in the 15 days prior to detection, both of whom came from the state of Mato Grosso, demonstrated in Fig. 3.

The most frequent symptoms of patients detectable for CHIKV were back pain (86.96%; 20/23), headache and arthritis (82.61%; 19/23), myalgia (52.17%; 12/23), retroorbital pain (47.83%; 11/23), nausea and severe arthralgia (39.13%; 9/23), rash (34.78%; 8/23), vomiting (21.74%; 5/23), conjunctivitis (17.39%; 4/23), and petechiae (13.04%; 3/23). Table 1 presents the association between the signs and symptoms observed in the studied population compared with positivity for the different arbovirus. In this analysis, it was possible to observe that the frequency of rash and conjunctivitis was significantly higher in individuals positive for CHIKV compared to individuals negative or positive for the other viruses identified in the study (*p* < *0.05)*.

**Table 1:** Comparison between clinical signs and symptoms and the detection of arboviral infections

Virus	Symptoms/Signs	n (%)	p-value	Odds Ratio (OR)	95% CI lower	95% CI upper
CHIKV	Headache	19 (82.6)	0.295	0.556	0.185	1.675
	Vomiting	5 (21.7)	0.558	0.742	0.272	2.024
	Back pain	20 (86)	0.271	1.965	0.577	6.688
	Arthritis	19 (82.6)	0.147	2.193	0.738	6.514
	Petechiae	3 (13)	0.411	1.838	0.531	6.366
	Myalgia	12 (52.2)	0.072	0.474	0.206	1.09
	Rash	8 (34.8)	0.0008*	5.425	2.222	13.243
	Nausea	9 (39.1)	0.226	0.595	0.255	1.392
	Conjunctivitis	4 (17.4)	0.0345*	3.805	1.238	11.698
	Severe Arthralgia	9 (39.1)	0.300	1.562	0.667	3.66
	Retro-orbital pain	11 (47.8)	0.421	0.712	0.311	1.634
DENV-1	Headache	7 (87.5)	0.590	0.82	0.1	6.748
	Vomiting	3 (37.5)	0.457	1.603	0.38	6.765
	Back pain	7 (87.5)	0.690	2.063	0.252	16.879
	Arthritis	4 (50)	0.272	0.462	0.115	1.861
	Petechiae	2 (25)	0.121	4.085	0.807	20.685
	Myalgia	7 (87.5)	0.446	3.044	0.372	24.879
	Rash	1 (12.5)	0.530	1.453	0.176	11.981
	Nausea	6 (75)	0.290	2.778	0.557	13.85
	Conjunctivitis	0 (0)	1	1.13	0.064	20.009
	Severe Arthralgia	1 (12.5)	0.449	0.347	0.042	2.837
	Retro-orbital pain	7 (87.5)	0.147	5.441	0.666	44.434
DENV-2	Headache	3 (100)	1	0.703	0.035	14.154
	Vomiting	0 (0)	0.566	0.445	0.022	8.925
	Back pain	3 (100)	1	1.768	0.088	35.464
	Arthritis	3 (100)	0.555	2.77	0.138	55.51
	Petechiae	0 (0)	1	2.042	0.101	41.254
	Myalgia	3 (100)	0.557	2.609	0.13	52.29
	Rash	1 (33.3)	0.247	5.086	0.455	56.793
	Nausea	0 (0)	0.112	0.154	0.008	3.091
	Conjunctivitis	0 (0)	1	3.012	0.148	61.132
	Severe Arthralgia	2 (66.6)	0.206	4.86	0.438	53.859
	Retro-orbital pain	3 (100)	0.261	4.664	0.233	93.412
MAYV	Headache	6 (100)	1	1.406	0.078	25.396
	Vomiting	1 (16.7)	1	0.534	0.062	4.6
	Back pain	4 (66.7)	0.624	0.589	0.107	3.244
	Arthritis	5 (83.3)	0.671	2.308	0.268	19.863
	Petechiae	0 (0)	1	1.021	0.056	18.507
	Myalgia	3 (50)	0.375	0.435	0.087	2.17
	Rash	2 (33.3)	0.097	5.086	0.915	28.261
	Nausea	1 (16.7)	0.112	0.185	0.022	1.593
	Conjunctivitis	0 (0)	1	1.506	0.083	27.429
	Severe Arthralgia	2 (33.3)	1	1.215	0.221	6.679
	Retro-orbital pain	4 (66.7)	0.701	1.555	0.283	8.537
OROV	Headache	3 (60)	1	0.703	0.035	14.154
	Vomiting	0 (0)	0.566	0.445	0.022	8.925
	Back pain	3 (60)	1	1.768	0.088	35.464
	Arthritis	2 (40)	1	0.923	0.083	10.231
	Petechiae	0 (0)	1	2.042	0.101	41.254
	Myalgia	2 (40)	1	0.87	0.078	9.638
	Rash	0 (0)	1	1.695	0.084	34.184
	Nausea	2 (40)	1	1.852	0.167	20.51
	Conjunctivitis	0 (0)	1	3.012	0.148	61.132
	Severe Arthralgia	2 (40)	0.206	4.86	0.438	53.859
	Retro-orbital pain	1	0.584	0.389	0.035	4.304

Legend: Association between clinical signs and symptoms and the detection of arboviral infections (DENV-1, DENV-2, CHIKV, MAYV, and OROV) in patients presenting with acute febrile illness lasting up to 7 days. Each virus-positive group was compared with samples negative for all investigated viruses. Statistical analyses were performed using the chi-square test or Fisher’s exact test, as appropriate according to expected cell frequencies. Odds ratios (OR) with 95% confidence intervals (95% CI) were calculated to estimate the strength of association. Values of p< 0.05 were considered statistically significant and are indicated with an asterisk (*). One patient detectable for DENV-1 was not included in the analysis due to lack of data

Sequencing was performed on 15 samples that tested molecularly positive for CHIKV by RT-qPCR. Phylogenetic analysis using the ML method demonstrated that all CHIKV samples analyzed (100%; 15/15) belonged to the East/Central/South African (ECSA) genotype (Fig. 4). The sequences showed a close genetic relationship with samples previously detected in other regions of Brazil. In the cluster containing the study samples, sequences were identified from both Alto Alegre dos Parecis, in the Southern Cone, and Porto Velho, in the Northern Cone of Rondônia, indicating intraregional spread following the introduction of the virus.

## Discussion

The results of this study provide a characterization of the CHIKV outbreak in the state of Rondônia, integrating epidemiological, molecular, and geographic aspects. These findings highlight the importance of arbovirus surveillance in the Western Amazon. The advancement of globalization and the intensification of migratory flows have contributed to the spread of pathogens in different regions of the world [29]. In this context, arboviruses have established themselves as a global public health problem, with a progressive increase in the number of cases and expansion into areas previously free of these infections [30]. In 2023, a territorial spread of the CHIKV virus was observed in Brazil, especially in the Southeast region, where the virus's circulation was previously concentrated in the Northeast region [31]. In 2024, it expanded to other regions, such as the Central-West region, especially the state of Mato Grosso, which experienced a significant increase in the number of Chikungunya cases [32].

In 2024, 155 cases of Chikungunya were recorded in the state of Rondônia. The following year, there was a significant increase of 1,842% in the number of confirmed cases compared to the previous year [33]. It is important to consider that the Southern Cone of the state of Rondônia borders states in the Central-West region, which were experiencing a Chikungunya outbreak. It is noteworthy that the increase in the number of cases occurred at the end of the year, a period characterized by intense population mobility due to festive celebrations. This scenario likely acted as a gateway for cases to enter Rondônia, favoring the introduction and subsequent circulation of the virus in the region. The analysis of the individuals revealed that 95% of the cases were negative for the arboviruses investigated in this study, even though all patients reported characteristic symptoms of acute febrile episodes at the time of care. As highlighted in other studies, factors such as the delay in seeking medical attention [31], regions of difficult access such as rural and riverside areas, as well as the possible circulation of other arboviruses and/or different pathogens in the region that are not diagnosed in routine laboratory testing, may have influenced the negative results observed in this study [32–34].

Regarding the sex of patients detectable for Chikungunya, there was no significant difference, diverging from previous published studies [35]. The symptoms observed in CHIKV-positive individuals do not differ from other studies, which frequently report high fever, arthralgia, headache, and myalgia, with intense arthralgia being the main complaint [34, 35],due to viral tropism, which causes intense inflammatory processes in the joints [38]. Despite the statistically significant difference observed in the frequency of rash and conjunctivitis compared to CHIKV-negative cases, it is important to emphasize that these signs are also present in other arboviruses such as DENV and ZIKV [39, 40] and are therefore not unique to this infection. Due to the analysis of only acute cases with up to 7 days of symptoms and the consequent lack of follow-up and monitoring of positive cases for a longer period, this study was unable to evaluate chronic symptoms associated with CHIKV, which can last for periods exceeding 12 weeks [41, 42]. The exclusive detection of the CHIKV ECSA genotype in the Rondônia samples is consistent with previous studies that indicate the predominance of this genotype in different Brazilian regions [43–45]. However, the Asian genotype was previously identified in 2014 in the city of Oiapoque, Amapá state (North region of the country), while the ECSA genotype was first identified in the same year in Feira de Santana, Bahia (Northeast region), and subsequently became predominant throughout the country to the present day [44, 46].

The phylogenetic similarity of the Rondônia samples to sequences from samples from different regions of the state suggests an introduction event followed by intraregional spread. Considering that immunity acquired after CHIKV infection tends to be long-lasting [47], the occurrence of outbreaks in Rondônia may be associated with the introduction of the virus into a previously susceptible population, with subsequent amplification of transmission.

This study presents some limitations that must be considered in the interpretation of the results. The clinical evaluation was limited to the acute phase of the infection, preventing the monitoring of progression to chronic manifestations. Moreover, the genomic analysis was conducted only on a partial region of the viral genome, preventing a more detailed spatiotemporal approach. Furthermore, the lack of entomological data, climate variables, and spatial modeling limits the integrated understanding of the ecological determinants of transmission in the Amazonian context, where factors such as vector density, rainfall seasonality, and population mobility play a central role in the dynamics of outbreaks.

## Conclusion

Epidemiological and molecular analysis revealed the spread of the Chikungunya virus in the state of Rondônia, as demonstrated by the increase in the number of cases and the characterization of the circulating genotype. These results contribute to understanding regional transmission dynamics and reinforce the need for integrated and continuous genomic surveillance to guide prevention and control measures for this arbovirus.

## Data Availability

The datasets supporting the conclusions of this article are included within the article and the access codes for the CHIKV sequences are deposited in Genbank under the identification PX471975-PX471989.
